# Gut microbiota transplantation for colonization of germ-free mice

**DOI:** 10.1016/j.xpro.2021.100610

**Published:** 2021-06-16

**Authors:** Jocelyn M. Choo, Geraint B. Rogers

**Affiliations:** 1 Microbiome and Host Health, South Australia Health and Medical Research Institute, Adelaide 5000, Australia; 2 Microbiome Research Laboratory, College of Medicine and Public Health, Flinders University, Bedford Park 5042, Australia

**Keywords:** Sequence analysis, Microbiology, Model Organisms, Molecular Biology

## Abstract

The use of germ-free mice is integral to the understanding of host-gut microbiome relationships. Such models rely on faithful replication of the donor microbiome to establish causal effects of the gut microbiota on host pathophysiology. This protocol describes the preparation and transfer of donor microbiota, focusing on strict anaerobic processing methods and multiple instillations by gavage for optimal gut microbiota recovery.

For complete details on the generation and use of this protocol, please refer to Choo and Rogers (2021).

## Highlights

• Describes preparation and transplantation of gut microbiota into germ-free mice
• Strict anaerobic processing of donor cecal microbiota is essential
• Faithful microbiome replication requires multiple instillations by gavage
• Adaptable to different gut materials and highlights recommended validation steps

## Graphical abstract

**Figure:**
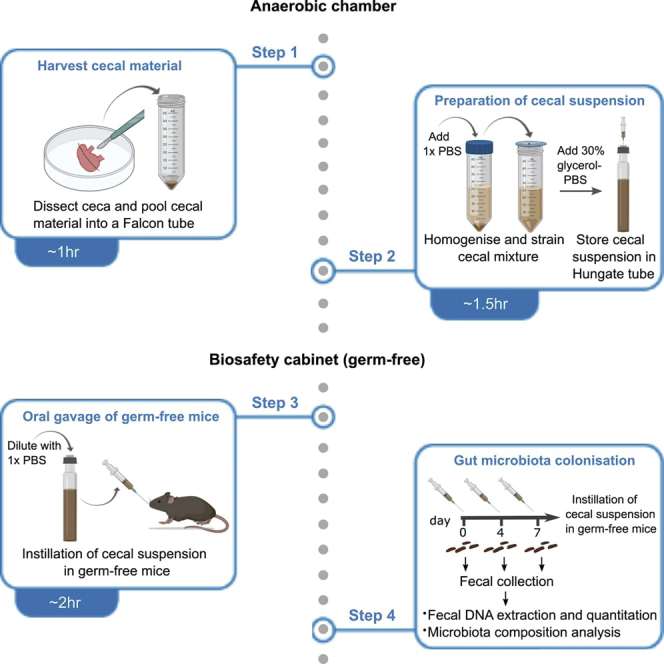

## Before you begin

### Reagent preparation

**Timing: 1 h preparation time, 10 days wait time**

1. Prepare sterile 30% glycerol in phosphate-buffered saline (PBS) solution (pH 7.4).
2. Prepare sterile 20% glycerol in PBS solution (pH 7.4).
3. Prepare sterile PBS solution (pH 7.4), stored in a Falcon tube.
4. Place all reagents in anaerobic chamber for at least 10 days prior to use.
5. Prepare sterile pre-reduced PBS solution (pH 7.4), stored in an anaerobic culture tube (for example, Hungate tube).
  a. Prepare sterilized Hungate tube as detailed in Figure 1.

**Figure 1:**
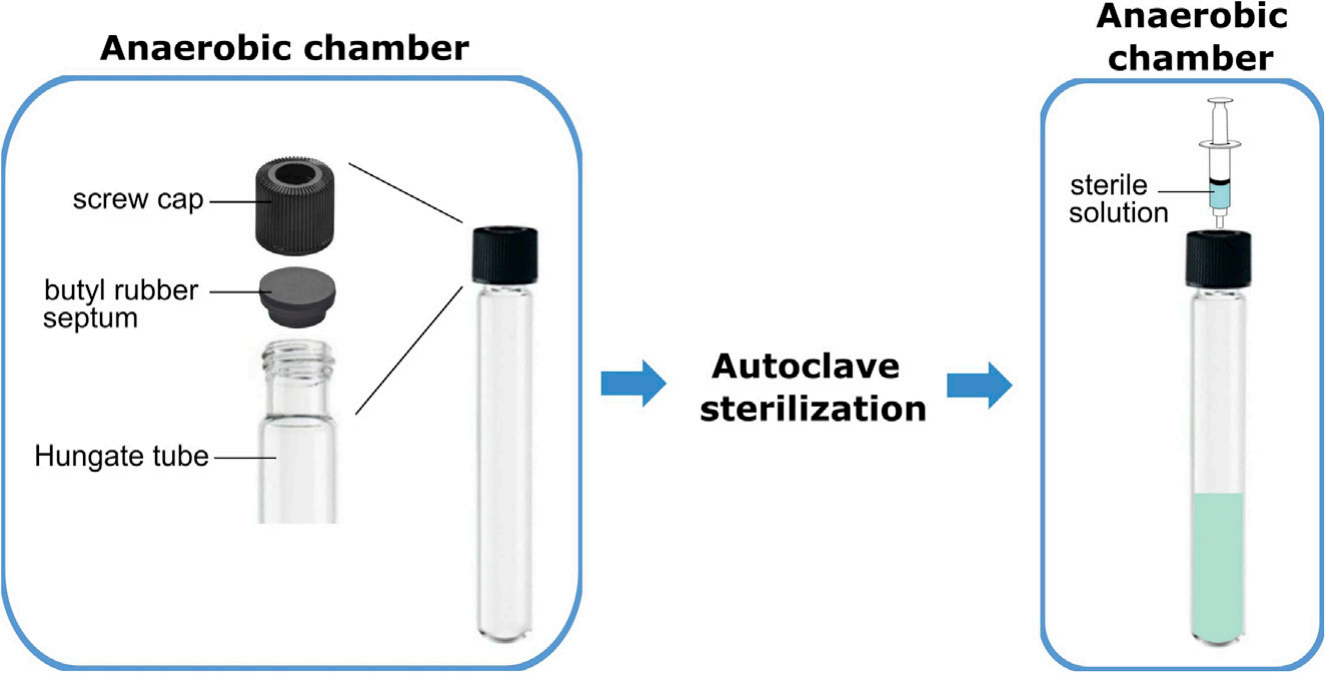
Preparation of Hungate tube Seal Hungate tube using a butyl rubber septum, followed by a screw cap in an anaerobic chamber. Sterilize the sealed Hungate tube by autoclaving. Following transport of the Hungate tube into an anaerobic chamber, transfer the appropriate solution into the Hungate tube using a sterile syringe and needle. Further information on the Hungate tube technique is accessible from the Leibniz Institute DSMZ (https://www.dsmz.de/fileadmin/Bereiche/Microbiology/Dateien/Kultivierungshinweise/Kultivierungshinweise_neu_CD/englAnaerob.pdf).
  b. In an anaerobic chamber, use a sterile syringe and 23G needle to transfer sterile pre-reduced PBS solution into Hungate tube.
6. Pre-weigh a sterile, capped, 50 mL Falcon tube to at least two decimal places (for use in step-by-step 5d).

***Alternatives:*** An anaerobic bag (for example, the Spilfyter Hands-in-Bag Atmospheric Chambers) may be considered as a cost-effective alternative to the anaerobic chamber to maintain an anaerobic environment. Equipment details are available from the supplier (https://us.vwr.com/store/product/4775838/spilfyter-hands-in-bag-atmospheric-chambers) and usage method is demonstrated in Weldy et al., 2020; Methods Video S1.

### Suitability of donor and recipient mice

**Timing: >10 days**

7. All animal procedures need to be approved by an Institutional Animal Care and Use Committee. All experiments in this protocol were approved by the South Australia Health and Medical Research Institute Animal Ethics Committee (SAM# 378).
8. Germ-free mice should be housed 2 to 5 animals of the same sex per cage, ensuring a random distribution of variables such as age and litter.
  a. House mice in a temperature-controlled room (22 ± 2°C) on a 12-h light/dark cycle.
9. Acclimatize mice to facility conditions for at least 10 days prior to inoculation.

***Note:*** Adult male mice should not be cohoused unless they were housed together before sexual maturity, which is reached between 40 to 60 days of age.

***Note:*** Factors that are known to contribute to variation in the gut microbiota include the sex (Elderman et al., 2018), genotype (Ericsson et al., 2017), diet (Rausch et al., 2016), and bedding type (Rausch et al., 2016). These variables should be kept consistent across donor mice, wherever possible.

***Note:*** This protocol was used for gut microbiota colonization of 6 to 7-week old female C57BL/6 germ-free mice using material from C57BL/6 donor mice. The protocol can be used for other adult donor or germ-free mouse sex, genotype and ages 6 weeks or older.

## Key resources table

**Table:** 

REAGENT or RESOURCE	SOURCE	IDENTIFIER
**Chemicals, peptides, and recombinant proteins**		

1× Phosphate-buffered saline (pH 7.4)	Thermo Fisher Scientific	10010
Glycerol	Thermo Fisher Scientific	17904

**Experimental models: Organisms/strains**		

Conventional mice	The Jackson Laboratory	C57BL/6
Germ-free mice	Translational Research Institute, Queensland, Australia	C57BL/6

**Other**		

Anaerobic chamber	COY	7150220
Hungate Tube (16 × 125 mm)	Bellco Glass	2047-16125
Corning 100 μm Cell Strainer	Corning	431752
Pestle for cell strainer	Sigma-Aldrich	Z742105
Petri dish 90 mm	Thermo Fisher Scientific	LBS60014X
Sterile spatula	Sigma-Aldrich	CLS3005-100EA
Disposable scalpel	Adelab Scientific	LV-SCP21
Syringe (1 mL, 10 mL, 30 mL)	BD	309659, 303134, 309651
Needle (23G)	Terumo	219912
Gavage needle	Instech	N/A
Minisart syringe filter (0.22 μm)	Sartorius	16532
Vortex mixer	Ratek	VM1-FT
QIAGEN DNeasy PowerLyzer PowerSoil kit	QIAGEN	12855-100
Qubit dsDNA HS Assay Kit	Thermo Fisher Scientific	Q32851
Qubit Flex Fluorometer	Thermo Fisher Scientific	Q33327
Tubes with screw cap (10 mL, 50 mL)	Thermo Fisher Scientific	348224, 339653
Teklad Global 18% Rodent Diet	Envigo	2018S

## Materials and equipment

**Table:** Pre-reduced 15% glycerol-phosphate buffered saline solution

Reagent	Final concentration	Amount
Glycerol	15%	7.5 mL
PBS (pH 7.4)	n/a	42.5 mL
**Total**	**Not applicable**	**50 mL**

**Table:** Pre-reduced 30% glycerol-phosphate buffered saline (PBS) solution

Reagent	Final Concentration	Amount
Glycerol	30%	15 mL
PBS (pH 7.4)	n/a	35 mL
**Total**	**Not applicable**	**50 mL**

***Note:*** All solution can be stored at room temperature (22 ± 2°C) for up to a year.

**CRITICAL:** The solution should be filter-sterilized into a sterile 100 mL bottle by passing the solution through a 0.22 μm pore size syringe filter using a syringe with luer lock, or an equivalent device with a similar pore size, such as a 0.2 μm sterile bottle top filter. A bottle with a large aperture is recommended to enable rapid reduction of the solution. The bottle containing the solution should be placed with the seal cap loosened in the anaerobic chamber at room temperature (22 ± 2°C) for at least 10 days prior to use. The mixture should be swirled at least once a day to aid complete reduction.

***Alternatives:*** Sterile PBS solution (pH7.4) can also be prepared in-house using the published protocol (Cold Spring Harbor Laboratory Press, 2006, https://doi.org/10.1101/pdb.rec8247).

## Step-by-step method details

### Harvest and preparation of pooled donor cecal material

**Timing: 2.5 h**

Preparation of cecal suspension under anaerobic conditions for inoculation into germ-free mice. The commensal gut microbiota is dominated by anaerobic bacteria. Processing of donor material under strict anaerobic conditions is therefore essential in maintaining viability across constituent taxa. The time duration indicated for this section is an approximate for processing the ceca of three to five mice of the same group.

**Figure 2:**
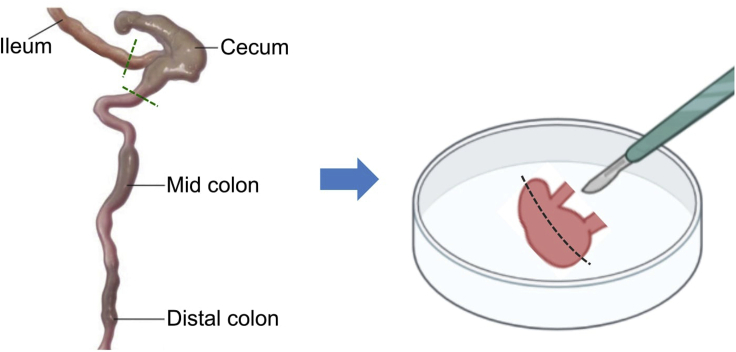
Dissection of cecum in donor mice Cecum is isolated from the gastrointestinal tract by performing a horizontal dissection at the junction between the cecum and the ileum, and between the cecum and colon, as indicated by the green dotted lines. The dissection section of the gastrointestinal tract should be within 0.5 cm of the cecum. A longitudinal incision is performed on the cecum (black dotted lines) for cecal material collection. Image of the mouse gastrointestinal tract is adapted from (Treuting et al., 2018).
4. Transfer cecum into a 10 mL Falcon tube containing 5 mL of pre-reduced 15% glycerol.
5. In an anaerobic chamber, place the cecum onto a sterile petri dish to harvest cecal material from the tissue encasing.
  a. Perform a longitudinal incision on the cecum using a sterile scalpel to expose cecal material.
  b. Use a sterile spatula to scrape and transfer cecal material into a pre-weighed empty 50 mL Falcon tube.
  c. Repeat this process for the remaining ceca from mice belonging to the same donor group.
  d. Pool all cecal material into the pre-weighed 50 mL Falcon tube.
6. Record weight of the 50 mL Falcon tube containing the cecal material and determine net weight.
7. Dilute cecal sample in 4× volume of pre-reduced PBS.
  a. For example, use 400 μL of PBS for every 100 mg cecum material.
8. Vortex cecal mixture for 1 min to obtain a homogeneous suspension.
  a. Use a pipette tip to disperse large pieces of material where necessary.
9. Place a 100 μm cell strainer onto a fresh sterile 50 mL Falcon tube (“suspension tube”).
10. Pipette the cecal material onto the cell strainer.
11. Allow the cecal suspension (supernatant) to flow into the 50 mL Falcon tube for 5 min.
  a. Use a sterile pestle to extract remaining supernatant in the cell strainer.
  b. Discard the cell strainer containing the remaining cecal material.
12. Dilute the cecal suspension with 1× volume of pre-reduced 30% glycerol.
13. Using a sterile syringe and 23G needle, draw up the cecal suspension from the “suspension tube” and transfer cecal suspension into a sealed Hungate tube.
  a. Aliquot the appropriate volume of cecal suspension into separate Hungate tubes.
  b. Allocate one Hungate tube per cage for each gavage day.
14. Store cecal suspension at −80°C until use.

***Note:*** Ideally, the dissected ceca in 15% glycerol should be processed immediately after harvest to obtain cecal suspension, in order to reduce freeze-thaw of cecal material. Freeze-thaw may affect the viability of bacteria as detailed in the limitations section. Where not possible, the dissected ceca can be stored in its entirety in 15% glycerol at −80°C, and preparation of cecal suspension should be performed as soon as possible.

***Note:*** Use a wide-bore tip when pipetting cecal material (steps 8 and 9) to prevent blockage. All material and reagents used should be sterile and aseptic technique practiced throughout.

1. Perform humane killing of mice according to ethics approval.
2. Position mouse in a supine position and perform a vertical midline incision to expose the gastrointestinal organs.
3. Dissect cecum from the gastrointestinal tract using a sterile scalpel (Figure 2).
  a. Perform a horizontal dissection at the junction between the cecum and ileum (0.5 cm from cecum).
  b. Perform a horizontal dissection at the junction between the cecum and colon (0.5 cm from cecum).

Anaerobic conditions should be maintained throughout the processing of donor material.

***Note:*** The total volume of cecal suspension to aliquot into separate Hungate tubes (step 13a) is calculated based on (n × 75 μL) + 200 μL. ‘n’ denotes the number of mice per cage. The addition of 200 μL to the total cecal suspension volume is to ensure sufficient volume of material for oral gavage per cage.

***Note:*** Pooled material from three ceca per group should provide sufficient material to perform the gut microbiota colonization step on 15 to 20 mice per group.

**Pause point:** Cecal suspension in Hungate tube can be kept frozen at −80°C until required for instillation into germ-free mice.

**CRITICAL:** Avoid repeated freeze-thaw of cecal suspensions to prevent loss of bacterial viability.

### Gut microbiota colonization of germ-free mice using cecal suspension

**Timing: 7 days**

Oral gavage of germ-free mice with cecal suspension is performed to achieve gut microbiota establishment.

15. Thaw Hungate tubes containing aliquots of the donor cecal suspension on ice on bench top (approximately 30 mins).
16. Transport germ-free mice, sterile pre-reduced PBS in Hungate tube, and cecal suspension in Hungate tube, into a sterilized biosafety cabinet in the germ-free animal facility.
17. Dilute cecal suspension in Hungate tube using 2× volume of sterile pre-reduced PBS.
  a. Add the required volume of PBS (in Hungate tube) into the Hungate tube containing cecal suspension using a sterile syringe and 23G needle.
  b. Vortex the diluted cecal suspension for 1 min.
18. Perform oral gavage using 150 μL of the cecal suspension according to institutional animal research facility safety operating practices (SOPs).
  a. Draw up cecal suspension using a sterile syringe and 23G needle.
  b. Replace 23G needle with a gavage needle for oral gavage.
19. Repeat steps 16–18 for oral gavage at day 4 and day 7.

***Optional:*** Collect fecal sample from individual mice prior to oral gavage at day 0, day 4, day 7, and appropriate time points thereafter for fecal DNA quantitation. Fresh fecal samples are collected using a sterile toothpick into a sterile 1.5-mL microcentrifuge tube. DNA extraction from fecal samples can be performed using the QIAGEN DNeasy PowerLyzer PowerSoil kit, or an equivalent DNA extraction kit. Quantitation of the extracted DNA can be performed using the Qubit dsDNA HS assay kit, or an equivalent fluorometric quantification assay. For high stringency bacterial DNA determination, quantitative PCR of the bacterial 16S rRNA gene can be performed on the extracted DNA samples, as described in Choo and Rogers, 2021.

***Optional:*** Gut microbiota composition of colonized germ-free mice can be determined using 16S rRNA amplicon sequencing to determine similarity to the donor gut microbiota, as described in Choo and Rogers, 2021.

***Note:*** Place thawed Hungate tube containing cecal suspension on ice on bench top until transported into the biosafety cabinet. Thawed cecal suspension can be kept at room temperature in the biosafety cabinet and should be used within 2.5h for oral gavage.

***Note:*** All procedures should be performed in a cleaned biosafety cabinet according to germ-free animal techniques. Prior to transfer of equipment into the biosafety cabinet, the external surfaces of all equipment should be sterilized using a suitable disinfectant solution according to animal facility SOPs. It is recommended to use one Hungate tube per cage to minimize the risk of contamination.

***Note:*** Where animal research facility SOP for oral gavage is not available, a reference for the procedure is available at https://ouv.vt.edu/content/dam/ouv_vt_edu/sops/small-animal-biomedical/sop-mouse-oral-gavage.pdf

## Expected outcomes

The cecal suspension preparation is expected to yield approximately 10^5^ – 10^6^ 16S rRNA copies/μL based on quantitative PCR of the 16S rRNA gene (Choo and Rogers, 2021).

**Figure 3:**
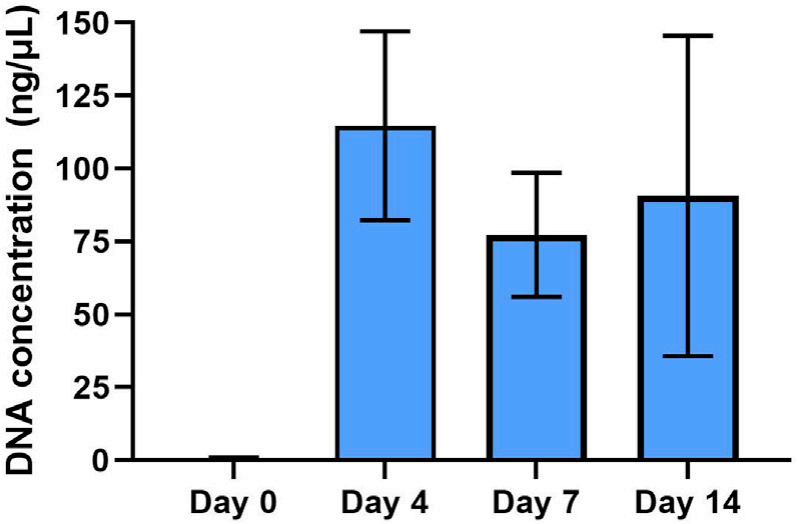
Concentration of DNA extracted from fecal samples of colonized germ-free mice DNA was extracted from fecal samples collected from colonized germ-free mice at baseline (day 0), day 4, day 7 and day 14 following the first gavage. The bar and error bars represent the mean and standard deviation, respectively.

Fecal bacterial load level of colonized germ-free mice is expected to reach the maximal average ± standard deviation of 96.0 ± 32.9 ng/μL by day 4 or day 7 (Figure 3). Detailed fecal bacterial load of colonized germ-free mice from day 0 to day 70 and those of the donor mice is indicated in Choo and Rogers, 2021; Figure 1. The dissimilarity/similarity of donor-recipient microbiota composition, and bacterial relative abundance in the recipient from day 4 to day 70 compared to the donor, is indicated in Figures 4 and 6, respectively, of Choo and Rogers, 2021.

## Limitations

Several factors may influence gut microbiota colonization of germ-free mice, including the age of germ-free mice, the gut material used for oral gavage, and the microbial community of donor mice. Previous microbiota colonization studies in conventional mice suggests that microbiota engraftment may be more effective when performed on younger mice (Le Roy et al., 2019). Multiple gavages into germ-free mice is expected to increase donor-recipient compositional similarity compared to those receiving only a single gavage (Eberl et al., 2020; Choo and Rogers, 2021), although this effect is dependent on the microbial community (Choo and Rogers, 2021). Given that engraftment of the transplanted microbiota is less efficient in a stable microbial community compared to a depleted intestinal microbiota environment (Le Roy et al., 2019), the inoculation of microbiota into germ-free mice during the early time points is expected to be more effective for microbiota colonization.

The method can be adapted for preparing gavage material from fecal samples or other sections of the gastrointestinal tract. However, optimization of the dilution method will need to be performed for these samples. It should be noted that fecal samples have larger exposure to atmospheric oxygen, which can reduce viability of bacteria (Papanicolas et al., 2019). These microbiota changes can reduce the efficiency of colonization and/or skew the recapitulation of the gut microbiota. In addition, the bacterial load and composition of the donor microbial community may be altered by interventions such as antibiotic treatment. Compositional differences of the gut microbiota can influence the efficiency of gut microbiota colonization in germ-free mice (Choo and Rogers, 2021). Where significant reduction in bacterial load is anticipated, the dilution factor should be optimized. The *in vitro* viability of murine microbiota were not affected in fecal samples that were frozen at −20°C for up to one month (Takahashi et al., 2018), although bacterial viability have been reported to reduce by up to half in freeze-thawed human fecal samples (Papanicolas et al., 2019). Therefore, cecal suspension should be frozen at −80°C and the number of freeze-thaw cycles minimized where possible.

## Troubleshooting

### Problem 1

Difficulty in pipetting cecal material due to blockage in pipette tip (step 10).

### Potential solution

Use a wide bore tip when pipetting cecal material. Disperse large pieces of material prior to pipetting suspension.

### Problem 2

Low volume recovery of cecal suspension (step 11).

### Potential solution

Use a pestle to dislodge cecal material blocking the cell strainer and to extract the remaining fluid. Use a new cell strainer if processing large volumes of cecal material.

### Problem 3

Low bacterial load levels in cecal suspension (step 13 and Expected Outcomes section).

### Potential solution

Where an intervention, such as antibiotic treatment, is expected to reduce the total gut bacterial load in donor mice, fecal samples should be collected from donor mice for DNA extraction and quantification prior to processing cecal material. The dilution factor and glycerol concentrations of cecal suspensions should be adjusted accordingly to achieve sufficient bacterial cells for oral gavage.

### Problem 4

Cecal suspension too viscous to pass through a plastic oral gavage needle (step 18).

### Potential solution

Reusable metal oral gavage needle that has been sterilized by autoclaving can be used to perform oral gavage of the cecal suspension. Personnel should be adequately trained and adhere to the SOP at all times.

### Problem 5

Cecum is not available for harvesting of inoculum material (step 3).

### Potential solution

Cecal material is used for the preparation of inoculum material as it yields a larger amount of material and has minimal exposure to oxygen. Where the cecum is unavailable for harvest, material from other regions of the lower gastrointestinal tract, such as the distal colon or feces, may be considered as a replacement to cecal material. Higher compositional similarity and microbiota stability is reported between the cecum, distal colon and feces, which microbiota composition differed to those of the small intestinal tract (Gu et al., 2013). However, the dilution method should be optimized and the constraints of using these material are indicated in the limitations section.

## Resource availability

### Lead contact

Further information and requests for resources and reagents should be directed to and will be fulfilled by the lead contact, Jocelyn Choo (jocelyn.choo@sahmri.com).

### Material availability

This study did not generate new unique reagents.

### Data and code availability

Original data for fecal bacterial load in colonized germ-free mice based on quantitative PCR of the 16S rRNA gene is available. [https://doi.org/10.1016/j.isci.2021.102049]
